# Body Adiposity in Later Life and the Incidence of Dementia: The Health in Men Study

**DOI:** 10.1371/journal.pone.0017902

**Published:** 2011-03-25

**Authors:** Brian D. Power, Helman Alfonso, Leon Flicker, Graeme J. Hankey, Bu B. Yeap, Osvaldo P. Almeida

**Affiliations:** 1 Department of Psychiatry, Royal Perth Hospital, Perth, Western Australia, Australia; 2 Western Australian Centre for Health and Ageing, Centre for Medical Research, University of Western Australia, Perth, Western Australia, Australia; 3 School of Psychiatry and Clinical Neurosciences, University of Western Australia, Perth, Western Australia, Australia; 4 School of Medicine and Pharmacology, University of Western Australia, Perth, Western Australia, Australia; 5 Department of Geriatric Medicine, Royal Perth Hospital, Perth, Western Australia, Australia; 6 Department of Neurology, Royal Perth Hospital, Perth, Western Australia, Australia; 7 Department of Endocrinology and Diabetes, Fremantle Hospital, Perth, Western Australia, Australia; McGill University/Douglas Mental Health University Institute, Canada

## Abstract

**Objective:**

To determine if adiposity in later life increases dementia hazard.

**Methods:**

Cohort study of 12,047 men aged 65–84 years living in Perth, Australia. Adiposity exposures were baseline body mass index (BMI), waist circumference (WC) and waist-to-hip ratio (WHR). We used the Western Australian Data Linkage System (WADLS) to establish the presence of new cases of dementia between 1996 and 2009 according to the International Classification of Diseases (ICD). Crude and adjusted hazard ratio (HR, 95% confidence interval, 95%CI) of dementia for each adiposity marker was calculated using Cox regression models. Other measured factors included age, marital status, education, alcohol use, smoking, diet, physical activity, and prevalent hypertension, diabetes, dyslipidaemia and cardiovascular disease.

**Results:**

Compared with men with BMI<25, participants with BMI between 25–30 had lower adjusted HR of dementia (HR = 0.82, 95% CI = 0.70–0.95). The HR of dementia for men with BMI≥30 was comparable to men with BMI<25 (HR = 0.82, 95%CI = 0.67–1.01). Waist circumference showed no obvious association with dementia hazard. Men with WHR≥0.9 had lower adjusted HR of dementia than men with WHR <0.9 (HR = 0.82, 95%CI = 0.69–0.98). We found a “J” shape association between measures of obesity and the hazard of dementia, with the nadir of risk being in the overweight range of BMI and about 1 for WHR.

**Conclusions:**

Higher adiposity is not associated with incident dementia in this Australian cohort of older men. Overweight men and those with WHR≥0.9 have lower hazard of dementia than men with normal weight and with WHR<0.9.

## Introduction

The prevalence of obesity in older Australians has tripled between 1985 and 2004, affecting 22% of men aged 65–74 and 14% of those older than 75 years [1]. However, there is controversy over whether obesity guidelines that have been developed for adults should be applied to the elderly [2], as mortality risk seems lowest for overweight older people [3]. Likewise, the association between measures of obesity and dementia risk remains debatable. Surveys investigating adiposity in mid- life indicate that being overweight or obese increases dementia risk later in life [4], [5], [6], [7], [8], [9], [10], although not all longitudinal data are supportive of such an association [11]. Observational studies examining the link between adiposity in later life and dementia risk have produced less consistent results. For example, in a 18-year prospective study in Sweden, higher body mass index (BMI) between ages 70 to 79 years increased the risk of dementia only in women aged 79–88 years [12]. In contrast, recent investigations found that older people who are overweight or obese may actually have lower risk of developing dementia than those who have normal weight [9], [13], [14], although confounding and healthy survivorship bias may explain these inconsistent and disparate findings.

The varying measures used to guide the definition of obesity represent another complicating factor when comparing studies of incident dementia, as some use BMI whilst others emphasize the importance of central obesity as determined by the waist circumference (WC) or waist-to-hip ratio (WHR).

We designed the present study to determine if BMI and measures of central adiposity are associated with incident dementia in a community-dwelling sample of older men, once demographic, lifestyle and medical morbidities are taken into account. As we used record linkage, loss to follow-up due to differential survival was negligible.

## Methods

### Study Design

Cohort study of men aged 65–84 years at the time of enrollment.

### Setting

We recruited community-dwelling men living in the Perth metropolitan area, Western Australia.

### Participants

**Figure 1:**
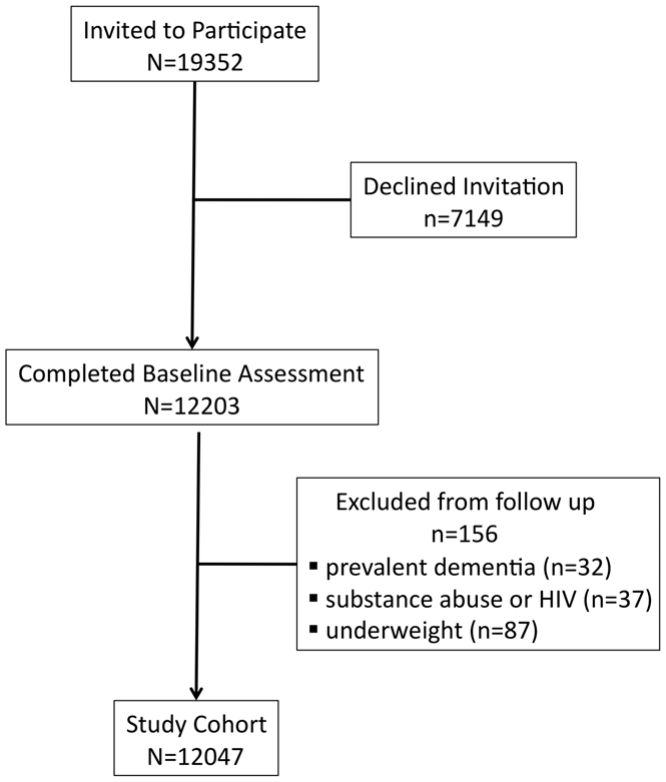
Flow of participants from invitation to inclusion in the study.

The Health In Men Study (HIMS) used a copy of the electoral roll to recruit participants in 1996 (voting is compulsory in Australia) [15]. Eligible men were aged between 65–84 and resident in Perth at the time of recruitment, were free of dementia and did not have mental or neurological disorders that could be attributed to the use of substances (such as alcohol) or HIV. We also excluded men with BMI<18.5 (Figure 1). After recruitment, participants were followed using the Western Australia Data Linkage System (WADLS) until they received a diagnosis of dementia, died or completed follow-up on the 30^th^ September 2009, whichever came first (see below). The Human Research Ethics Committee of the University of Western Australia approved the study protocol and all participants offered written informed consent. This project was conducted according to the principles of the Declaration of Helsinki.

### Outcomes of interest

Morbidity and mortality data were retrieved from WADLS, one of the most comprehensive electronic health record systems in the world [16]. WADLS brings together name-identified records for all in-patient hospital admissions as well as public sector mental health services (in-patient, out-patient and community mental health services), and includes all morbidity and mortality data of Western Australia coded according to the International Classification of diseases tenth revision (ICD-10) and, for events that occurred before 1996, the ICD-9.

The diagnosis of dementia was the primary endpoint of interest of the study, and was defined according to the following ICD-9 and ICD-10 codes: all listed diagnoses (primary and secondary) for Alzheimer's dementia 331.0, F00, G30; Vascular dementia 290.4, F01; fronto-temporal dementia 331.1, F02.0, G31.0; Huntington's disease 333.4, G10, F02.2; Parkinson's dementia or dementia with Lewy bodies F02.3, 331.82; and non-specific dementia 290.0, 290.1, 290.2, 290.3, 290.8, 290.9, 294.1, 294.8, 331.2, F02.8, F03, F05.1, G31.1, G31.8, G31.9. To improve case ascertainment, the text terms of the above conditions were searched for in the morbidity and mortality data systems, along with the following alternative medical terms: multi-infarct dementia, arteriosclerotic dementia, fronto-temporal lobe dementia, primary progressive dementia, corticobasal dementia, and Pick's dementia. As the accuracy of specific diagnostic causes of dementia in WADLS is uncertain, we opted to group all entries under the general heading of ‘dementia’.

### Adiposity measures

We used standard procedures to measure weight (to 0.2 Kg) and height (to 0.5 cm) of all participants. We calculated the BMI in kg/m^2^ and classified participants as normal weight (18.5≤BMI<25), overweight (25≤BMI<30) and obese (BMI≥30). We used a steel measuring tape to determine the WC in centimeters, with measurements taken halfway between the lower border of the ribs and the iliac crest in a horizontal plane (to 0.5 cm). A similar procedure was used to measure hip circumference, with measurements taken at the widest point around the left and right greater trochanters. Men were wearing light clothing at the time of these measurements, which followed the recommendations of the World Health Organization [17]. We classified men with 94 cm≤WC<102 cm as having mild central obesity, and those with WC≥102 cm as having marked central obesity [18]. Similarly, we considered that a WHR≥0.9 indicated the presence of obesity [19].

### Other measures

We used a self-rating questionnaire to collect demographic, lifestyle and clinical information from participants; factors chosen for analysis were those which have previously been shown in the literature to be related to cognitive decline in older men. We calculated the age of participants by subtracting the date of their birth from the date of their assessment. We grouped men according to whether they had been born in Australia or overseas, and recorded men as married if they reported being married or were living in a de facto relationship. In addition, participants recorded their highest educational achievement (completed high school education or greater vs did not complete high school education). Lifestyle data included smoking history (never smoked, past smoker, current smoker), alcohol intake reported as standard drinks in a usual week (none, less than 28 standard drinks per week, 28 or more standard drinks per week), vigorous exercise of at least 150 minutes per week (e.g., jogging), and non-vigorous exercise (e.g., walking); questions regarding eating habits were limited to the number of times per week participants consumed meat and fish, as well as the type of milk they used (e.g., condensed, full cream, reduced-fat milk intake only, or a mixture of these). Information about premorbid clinical diseases and treatment were self-reported at the time of recruitment into the study. We asked participants, “have you ever been told by a doctor you suffer from or have you ever received treatment for” diabetes (yes/no), dyslipidemia (high cholesterol or triglycerides) (yes/no), hypertension (yes/no) or coronary heart disease (if the patient reported having been told by a doctor that they had suffered a heart attack or that they had angina) (yes/no).

### Statistical analysis

We used Cox proportional hazards regression models to determine the univariate association between measured demographic, lifestyle and clinical variables with incident dementia. Survival time was calculated from the date of entry into the study to the date of first recorded diagnosis of dementia, date of death, or the end of follow-up (September 30, 2009), whichever came first. We then investigated the association between markers of adiposity (BMI, WC, WHR) and incident dementia using, again, Cox proportional hazard models, and later adjusted those analyses for possible confounding factors identified in our initial univariate analysis (p<0.15), as described above. The cumulative hazard of dementia over follow-up was determined and presented graphically according to categories for each adiposity marker. We also investigated the association between dementia and each adiposity marker by using a restricted cubic spline with 3 knots (i.e., a function that produces a smooth curve fitted statistically through three given data points, allowing more flexibility in the regression model and the investigation of non-linear associations [20]) and modeling each adiposity marker as a continuous variable; results from these analyses are shown graphically. Finally, to examine the robustness of our results, we re-ran all analyses after excluding men with a survival time of less than 2 years, either because they developed dementia or died during the first two years of follow-up. The annual rate of dementia according to the relevant exposures was estimated using the Stata command *strate*. Ninety-five percent confidence intervals (95%CI) were estimated for the HR and rates. Alpha was set at 5% and all probability tests reported are two-tailed. We used Stata version 11.1 (StataCorp, College Station, Texas) for data management and analysis.

## Results

Twelve thousand and forty-seven men were included in the study. Their age ranged from 65 to 84 years at the time of entry (mean±SD = 72.1±4.4) and 1,271 developed dementia during the 9.7±3.5 (range: 0.02 to 13.4) years of follow-up; those with dementia had a mean time in the study of 7.1±3.0 years, and those without dementia 10.0±3.6 years (difference due to censoring because follow-up was discontinued once the diagnosis of dementia was established). The average annual rate of dementia in this cohort was 10.9% (95%CI = 10.3–11.5).

Table 1 shows the distribution of demographic, lifestyle and clinical variables amongst study participants. Univariate Cox regression showed that the hazard of dementia increased with age, but was lower amongst those who were married or had completed high school. Moderate alcohol use was associated with lower hazard of dementia compared with no alcohol use, as was the consumption of reduced-fat milk and vigorous physical activity. Men with diabetes, dyslipidemia and coronary heart disease had a greater hazard of dementia than men without these conditions.

**Table 1:** Demographic, lifestyle and past medical characteristics of participants at time of entry into the study and according to dementia outcome.

		NO DEMENTIA	DEMENTIA	
		N = 10,776	N = 1,271	
BASELINE CHARACTERISTIC		N (%)	N (%)	HR (95%CI)
**Age (years)**	65–69	4362 (40.5)	249 (19.6)	1 (reference)
	70–74	3802 (35.3)	409 (32.2)	2.02 (1.73–2.36)
	75–79	2074 (19.2)	444 (34.9)	4.48 (3.84–5.24)
	≥80	538 (5.0)	169 (13.3)	7.59 (6.24–9.24)
**Migrated**		4862 (45.1)	552 (43.4)	0.94 (0.84–1.05)
**Married**		8816 (81.8)	977 (76.9)	0.68 (0.60–0.78)
**Completed high school**		4373 (40.6)	465 (36.6)	0.81 (0.72–0.91)
**Smoking**	Never	3147 (29.2)	382 (30.1)	1 (reference)
	Past	6455 (59.9)	756 (59.5)	1.05 (0.93–1.19)
	Current	1169 (10.8)	133 (10.5)	1.12 (0.92–1.37)
**Alcohol use (SD)**	None/sporadic	1704 (15.8)	230 (18.1)	1 (reference)
	<28 SD/week	6175 (57.3)	653 (51.4)	0.76 (0.65–0.88)
	≥28 SD/week	813 (7.5)	96 (7.6)	0.88 (0.69–1.12)
**Diet**	Meat <2/week	2673 (24.8)	324 (25.5)	1.03 (0.90–1.16)
	Fish>2/week	1101 (10.2)	137 (10.8)	1.05 (0.88–1.26)
	Reduced-fat milk	3499 (32.5)	376 (29.6)	0.86 (0.76–0.97)
**Physical activity**	No exercise	2670 (24.8)	349 (27.5)	1 (reference)
	Non vigorous	5324 (49.4)	673 (53.0)	0.90 (0.79–1.02)
	Vigorous	1002 (9.3)	112 (8.8)	0.73 (0.59–0.90)
**Hypertension**		4513 (41.9)	547 (43.0)	1.12 (1.00–1.25)
**Diabetes**		1216 (11.3)	187 (14.7)	1.51 (1.29–1.76)
**Dyslipidaemia**		3327 (30.9)	305 (24.0)	1.45 (1.27–1.65)
**Coronary heart disease**		2669 (24.8)	361 (28.4)	1.36 (1.20–1.53)

HR = hazard ratio; 95%CI = 95% confidence interval of the hazard ratio; SD = standard drinks.

### Measures of adiposity and dementia risk

Table 2 summarizes the association between measures of adiposity and incident dementia. Overweight men had lower dementia hazard than men with normal BMI. Similarly, men with WHR≥0.9 had lower hazard of dementia than men with WHR<0.9. We then investigated if adiposity measures interacted with age to mediate the hazard of dementia amongst participants: the interaction terms between age and all adiposity measures were not significant (p>0.1; data not shown).

**Table 2:** Association between measures of adiposity and incident dementia.

		NO DEMENTIA	DEMENTIA	ANNUAL RATE OF DEMENTIA	HAZARD OF DEMENTIA	
ADIPOSITY MEASURES		N (%)	N (%)	per thousand (95% CI)	crude HR (95% CI)	adjusted HR (95% CI)
**BODY MASS INDEX (BMI) kg/m^2^**	BMI<25	3256 (30.2)	450 (35.4)	12.9 (11.8–14.2)		
	25≤BMI<30	5546 (51.1)	607 (47.8)	10.1 (9.2–10.8)	**0.76 (0.67–0.86)**	**0.82 (0.70–0.95)**
	BMI≥30	1966 (18.2)	214 (16.8)	10.1 (8.8–11.5)	**0.77 (0.66–0.91)**	0.82 (0.67–1.01)
**WAIST CIRCUMFERENCE (WC)**	WC<94 cm	3481 (32.3)	437 (34.4)	11.6 (10.5–12.7)		
	94.0≤WC<102 cm	3443 (32.0)	422 (33.2)	11.1 (10.1–12.2)	0.96 (0.84–1.10)	1.02 (0.87–1.20)
	WC≥102 cm	3848 (35.7)	412 (32.4)	10.1 (9.1–11.1)	**0.88 (0.77–1.00)**	0.88 (0.74–1.04)
**WAIST-TO-HIP RATIO (WHR)**	WHR<0.9	1695 (15.7)	236 (18.6)	12.6 (11.1–14.3)		
	WHR≥0.9	9076 (84.2)	1035 (81.4)	10.6 (9.9–11.2)	**0.84 (0.73–0.97)**	**0.82 (0.69–0.98)**

HR =  hazard ratio; 95%CI = 95% confidence interval of the hazard ratio.

Adjusted HR = hazard ratio adjusted for age, marital status, education, alcohol consumption, fat intake from milk, physical activity, and prevalent diabetes, dyslipidaemia and coronary heart disease.

**Figure 2:**
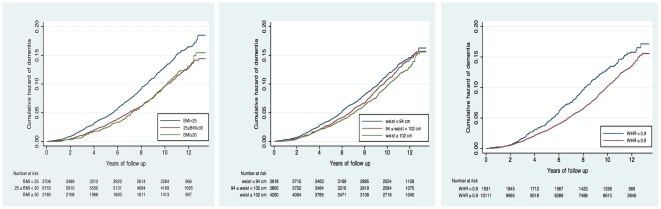
Cumulative hazard of dementia over time according to adiposity markers in elderly men. The left, middle and right panels show the hazard ratio (HR) of dementia over time for body mass index (BMI), waist circumference (WC) and waist-to-hip ratio (WHR) groupings, respectively.

Figure 2 shows the cumulative hazard of dementia over time for BMI (panel A), WC (panel B) and WHR (panel C) groupings. Cumulative hazard of dementia over the study period was highest for those with normal BMI compared with men who were overweight or obese. Similarly, the cumulative hazard of dementia was higher for those with WHR<0.9 than for men with WHR>0.9. The cumulative hazard of dementia did not change substantially with WC measures.

**Figure 3:**
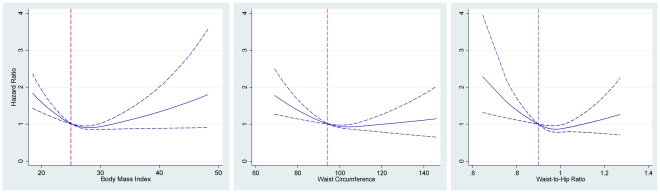
Average hazard of dementia (blue line) during follow-up according to the body mass index (BMI) (left panel), waist circumference (WC) (middle panel) and waist-to-hip ratio (WHR) (right panel). The vertical dashed red line represents the reference value for each indicator: 25 for BMI, 94 cm for WC, and 0.9 for WHR. The dashed blue lines represent the 95% confidence interval of the hazard ratio.

Figure 3 shows the association between BMI (panel A), WC (panel B) and WHR (panel C) measurements and the hazard of dementia in older men. A “J” curve association between measures of adiposity and hazard of dementia was seen, with the nadir of risk being in the overweight range of BMI, around 100 cm of WC and just under 1 in WHR. The widening confidence limits of the hazard of dementia associated with the extremes of adiposity measures makes relative comparisons within each group difficult.

### Sensitivity analysis

We re-ran all analyses after the exclusion of men who developed dementia (n = 68) or died (n = 480) during the first 2 years of follow-up. The results remained unchanged. Men with BMI between 25.0 and 30 had lower HR of dementia compared with normal weight men (crude HR = 0.78, 95%CI = 0.69–0.88; adjusted HR = 0.82, 95%CI = 0.70–0.95). The results for men with BMI≥30 remained borderline non-significant (crude HR = 0.80, 95%CI = 0.68–0.95; adjusted HR = 0.84, 95%CI = 0.69–1.03). There was no obvious association between WC grouping and dementia hazard (data not shown), but men with WHR≥0.9 continued to have lower HR of dementia than men with WHR < 0.9 (crude HR = 0.84, 95%CI = 0.73–0.97; adjusted HR = 0.81, 95%CI = 0.68–0.98).

## Discussion

The results of our study show that body mass index and waist-to-hip ratio measurements are associated with incident dementia risk in community-dwelling older men. We found that overweight men (25≤BMI<30) and those with WHR≥0.9 had the lowest dementia hazard over 10 years of follow-up, which suggests that guidelines of body composition used in mid-life should not be used for older men when evaluating their risk of dementia.

### Limitations of the study design

HIMS is an observational study and the distribution of exposures did not occur at random. In addition, our sample included only men and we are therefore unable to comment on whether these findings would apply equally to women. We also acknowledge that differential survival could have biased the results of the study, as men who were overweight or obese in mid-life might have died of competing diseases before reaching older age [21], so that surviving overweight and obese older men might be unusually healthy compared with their normal weight counterparts. We have previously reported that HIMS participants have lower mortality hazard than men who were invited but chose not to participate, and men in the general population who were not invited [22]. This suggests that HIMS participants are healthier than other men of their age living in the community, so that caution is required when attempting to generalize our findings. Furthermore, HIMS recruited men who were living independently in the community, thereby limiting the inclusion of people with overt dementia in the sample (hence the low prevalence of dementia in the inception cohort).

Data on prevalent health conditions was obtained by self-report (i.e., medical conditions present at the time of recruitment), and the external validity of such data is yet to be established. Another factor to consider is that the diagnosis of dementia in our study relied on the use of data from administrative records and less severe cases of dementia could conceivably not have been identified. Such a bias would only influence the results of the study if the distribution of error across the obesity groups was not random – for example, if normal weight men with dementia were greater users of health services than their overweight or obese counterparts. This seems an unlikely explanation for our results, as overweight and obese people use of health services more frequently than people with normal weight (i.e., the opportunity to make the diagnosis of dementia would be greater for overweight or obese people because they have more frequent contacts with the health services) [23]. Moreover, the observed prevalence of dementia in our sample (with 10.3% (1,271/12047) developing dementia during follow-up) is similar to those reported by others using different case-ascertainment methodologies [9], [13], [14]. Another limitation of our study is the uncertain validity of the specific diagnosis of dementia using record linkage. For this reason, we did not attempt to investigate the association between obesity and the varying causes of dementia and limited our analyses to the syndromal grouping of ‘dementia’. BMI, WC and WHR measurements were available only at the time of entry into the study and, consequently, we cannot comment on whether repetitive measures would have been more informative in predicting the development of dementia during follow-up.

It is also possible that obesity measures are associated with differential survival and that this may have confounded their association with dementia. We attempted to address this issue by excluding from the analysis men who died during the initial 2 years of follow-up, which did not alter the results of the study. We could have extended this exclusion to men who died within the first 5 years of follow-up, but this would have reduced the power of the study substantially in such an old cohort. In fact, our group has previously shown that survival amongst these old men was longest for those in the overweight group [3]. As overweight men survive longer, they would have greater opportunity of receiving the diagnosis of dementia during follow-up, which is exactly the opposite of what we observed (i.e., our findings seem robust).

There is also some evidence that older people who are underweight have a higher risk of dementia than those with normal weight [9], [24], but the association between low BMI and increased dementia risk seems to be due to acute loss rather than longstanding low body mass [9], [24]. Others have reported similar findings in younger age groups [9], [14]. For this reason, men with BMI in the underweight range were excluded from our analyses and, despite this conservative approach, we found that low adiposity in the normal range seems to increase the 10-year risk of incident dementia in older men. Some authors suggest that rather than being a risk factor for dementia, low BMI may be a marker of an underlying dementia process not yet clinically detectable [9], [24], and Atti and colleagues (2008) postulated that the time-dependent association they observed in their study supported this possibility. This study attempted to address the issue of reverse causality by excluding those people with a survival time of less than 2 years (either because they developed dementia or died), and this did not alter the results. As previously mentioned, a limitation of the study was having gathered adiposity measures at only one point, hence we were unable to monitor any changes in the weight of participants over time.

This study has strengths that also merit comment. To the best of our knowledge, this is one of the largest and longest studies of older men involved in an investigation of adiposity and dementia in later life. Further, rather than being restricted to BMI like many previous studies investigating the relationship between adiposity and dementia, HIMS collected information on three key-markers of adiposity, two of which are considered good indicators of central obesity and are more strongly associated with cardiovascular endpoints than BMI [25], [26]. Bias due to attrition is another important issue to consider in longitudinal studies; however in HIMS follow-up was assured as outcomes were monitored via the WADLS and less than 1% of older Western Australians die outside the state [27]. Moreover, our sensitivity analysis showed that the results of the study remained consistent after the exclusion of men who developed dementia or died during the first 2 years of follow-up. However, we concede that some men may have had incipient dementia at the time of recruitment that only became clinically apparent after 2 years, and as a result the possibility of reverse causality cannot be dismissed entirely.

### Interpretation of findings

Our results suggest that measures of adiposity are associated with incident dementia in later life, with the lowest dementia hazard falling within the overweight BMI range and WHR≥0.9. The cumulative hazard of dementia was greatest amongst normal weight men (BMI<25) and those with WHR<0.9. We also found a J-shaped association between dementia hazard and measures of adiposity, with nadir in the overweight range, which suggests that mild to moderate accumulation of adipose tissue reduces the risk of dementia in older men. These findings have relevance to the ongoing debate about developing suitable guidelines for adiposity markers in later life: older men who have a slight increase in adiposity markers may not be at greater risk of dementia, and current guidelines for “healthy” adiposity values for BMI, WC and WHR might require recalibration in older age [3].

Numerous studies have shown that excess abdominal fat (caused largely by the accumulation of visceral fat) correlates with an increased risk of adverse health outcomes, such as hypertension, dyslipidemia and hyperglycemia [28], [29], and as a measure of abdominal visceral fat, WC appears to be a more sensitive indicator of adverse health outcomes than BMI or WHR [25], [26], [30]. One way of explaining these findings is to consider that BMI and WHR, but not WC, may be indicators of physiological and nutritional fitness in later life. It is possible, for example, that changes in the quantity and distribution of adipose tissue in older age are adaptive and may confer a protective effect to the brain that minimizes the risk of cognitive decline, and that the loss of adipose tissue may deprive the aging brain of that benefit [31]. At this stage such an explanation is highly speculative, but evidence linking overweight to decreased mortality in older age suggests that this hypothesis cannot be simply dismissed [3]. Furthermore, preliminary basic science data suggests that our findings may be biologically plausible. For example, leptin, which is produced in the adipose tissue, is thought to be involved in brain development, learning and memory [32]. Direct administration of leptin into the hippocampus in mice improves memory processing and increases the expression of NMDA receptors, enhances learning and memory performance, and contributes to regulate hippocampal synaptic plasticity [33]. Interestingly, the serum concentration of leptin shows an inverse relationship with the risk of Alzheimer's disease in later life [34], [35].

Nonetheless, our results are also consistent with those of recent prospective studies indicating that whilst mid-life obesity is a risk factor for dementia, overweight and mild obesity in later life are not [9], [13], [14]. Nonetheless, disparate results have also been reported, particularly for older women [12], [36], and reasons for these conflicting outcomes have been discussed at length by others [37].

In conclusion, BMI and WHR measurements have a J-shaped association with dementia risk in this Australian cohort of older men, with the lowest risk falling within the overweight range. If replicated, these findings would have implications for public health recommendations on adiposity measures and for research into the physiological mechanisms linking adiposity to dementia in later life.

## References

[1] Australian Institute of Health and Welfare (2004). Obesity trends in Older Australians..

[2] Heiat A, Vaccarino V, Krumholz HM (2001). An evidence-based assessment of federal guidelines for overweight and obesity as they apply to elderly persons.. Arch Intern Med.

[3] Flicker L, McCaul KA, Hankey GJ, Jamrozik K, Brown WJ (2010). Body mass index and survival in men and women aged 70 to 75.. J Am Geriatr Soc.

[4] Kivipelto M, Ngandu T, Fratiglioni L, Viitanen M, Kareholt I (2005). Obesity and vascular risk factors at midlife and the risk of dementia and Alzheimer disease.. Arch Neurol.

[5] Rosengren A, Skoog I, Gustafson D, Wilhelmsen L (2005). Body mass index, other cardiovascular risk factors, and hospitalization for dementia.. Arch Intern Med.

[6] Whitmer RA, Gunderson EP, Barrett-Connor E, Quesenberry CP, Yaffe K (2005). Obesity in middle age and future risk of dementia: a 27 year longitudinal population based study.. BMJ.

[7] Whitmer RA, Gustafson DR, Barrett-Connor E, Haan MN, Gunderson EP (2008). Central obesity and increased risk of dementia more than three decades later.. Neurology.

[8] Whitmer RA, Sidney S, Selby J, Johnston SC, Yaffe K (2005). Midlife cardiovascular risk factors and risk of dementia in late life.. Neurology.

[9] Fitzpatrick AL, Kuller LH, Lopez OL, Diehr P, O'Meara ES (2009). Midlife and late-life obesity and the risk of dementia: cardiovascular health study.. Arch Neurol.

[10] Gustafson DR, Backman K, Waern M, Ostling S, Guo X (2009). Adiposity indicators and dementia over 32 years in Sweden.. Neurology.

[11] Stewart R, Masaki K, Xue QL, Peila R, Petrovitch H (2005). A 32-year prospective study of change in body weight and incident dementia: the Honolulu-Asia Aging Study.. Arch Neurol.

[12] Gustafson D, Rothenberg E, Blennow K, Steen B, Skoog I (2003). An 18-year follow-up of overweight and risk of Alzheimer disease.. Arch Intern Med.

[13] Atti AR, Palmer K, Volpato S, Winblad B, De Ronchi D (2008). Late-life body mass index and dementia incidence: nine-year follow-up data from the Kungsholmen Project.. J Am Geriatr Soc.

[14] Luchsinger JA, Patel B, Tang MX, Schupf N, Mayeux R (2007). Measures of adiposity and dementia risk in elderly persons.. Arch Neurol.

[15] Norman PE, Flicker L, Almeida OP, Hankey GJ, Hyde Z (2009). Cohort Profile: The Health In Men Study (HIMS).. Int J Epidemiol.

[16] Holman CD, Bass AJ, Rouse IL, Hobbs MS (1999). Population-based linkage of health records in Western Australia: development of a health services research linked database.. Aust N Z J Public Health.

[17] World Health Organisation (2000). Obesity: preventing and managing the global epidemic. Report of a WHO consultation.. World Health Organ Tech Rep Ser.

[18] Klein S, Allison DB, Heymsfield SB, Kelley DE, Leibel RL (2007). Waist circumference and cardiometabolic risk: a consensus statement from shaping America's health: Association for Weight Management and Obesity Prevention; NAASO, the Obesity Society; the American Society for Nutrition; and the American Diabetes Association.. Diabetes Care.

[19] World Health Organisation (1999). Definition, diagnosis and classification of diabetes and its complications.. Part 1:diagnosis and classification of diabetes mellitus provisional report of a WHO consultation.

[20] Heinzl H, Kaider A (1997). Gaining more flexibility in Cox proportional hazards regression models with cubic spline functions.. Comput Methods Programs Biomed.

[21] Pischon T, Boeing H, Hoffmann K, Bergmann M, Schulze MB (2008). General and abdominal adiposity and risk of death in Europe.. N Engl J Med.

[22] Norman PE, Jamrozik K, Lawrence-Brown MM, Le MT, Spencer CA (2004). Population based randomised controlled trial on impact of screening on mortality from abdominal aortic aneurysm.. BMJ.

[23] Quesenberry CP, Caan B, Jacobson A (1998). Obesity, health services use, and health care costs among members of a health maintenance organization.. Arch Intern Med.

[24] Nourhashemi F, Deschamps V, Larrieu S, Letenneur L, Dartigues JF (2003). Body mass index and incidence of dementia: the PAQUID study.. Neurology.

[25] Janssen I, Katzmarzyk PT, Ross R (2004). Waist circumference and not body mass index explains obesity-related health risk.. Am J Clin Nutr.

[26] Zhu S, Heshka S, Wang Z, Shen W, Allison DB (2004). Combination of BMI and Waist Circumference for Identifying Cardiovascular Risk Factors in Whites.. Obes Res.

[27] Bradshaw PJ, Jamrozik K, Jelfs P, Le M (2000). Mobile Australians: a moving target for epidemiologists.. Med J Aust.

[28] Okosun IS, Cooper RS, Rotimi CN, Osotimehin B, Forrester T (1998). Association of waist circumference with risk of hypertension and type 2 diabetes in Nigerians, Jamaicans, and African-Americans.. Diabetes Care.

[29] Boyko EJ, Fujimoto WY, Leonetti DL, Newell-Morris L (2000). Visceral adiposity and risk of type 2 diabetes: a prospective study among Japanese Americans.. Diabetes Care.

[30] Rankinen T, Kim SY, Perusse L, Despres JP, Bouchard C (1999). The prediction of abdominal visceral fat level from body composition and anthropometry: ROC analysis.. Int J Obes Relat Metab Disord.

[31] Harvey J, Shanley LJ, O'Malley D, Irving AJ (2005). Leptin: a potential cognitive enhancer?. Biochem Soc Trans.

[32] Davidson TL, Kanoski SE, Walls EK, Jarrard LE (2005). Memory inhibition and energy regulation.. Physiol Behav.

[33] Harvey J, Solovyova N, Irving A (2006). Leptin and its role in hippocampal synaptic plasticity.. Prog Lipid Res.

[34] Power DA, Noel J, Collins R, O'Neill D (2001). Circulating leptin levels and weight loss in Alzheimer's disease patients.. Dement Geriatr Cogn Disord.

[35] Lieb W, Beiser AS, Vasan RS, Tan ZS, Au R (2009). Association of plasma leptin levels with incident Alzheimer disease and MRI measures of brain aging.. JAMA.

[36] Hayden KM, Zandi PP, Lyketsos CG, Khachaturian AS, Bastian LA (2006). Vascular risk factors for incident Alzheimer disease and vascular dementia: the Cache County study.. Alzheimer Dis Assoc Disord.

[37] Luchsinger JA, Gustafson DR (2009). Adiposity and Alzheimer's disease.. Curr Opin Clin Nutr Metab Care.

